# Impact of inter-twin growth discordance on preeclampsia: based on ultrasonic estimated fetal weight

**DOI:** 10.1038/s41440-024-02027-5

**Published:** 2024-12-04

**Authors:** Lan Chen, Zhijuan Cao, Ping Qiao, Xiaohua Liu, Hao Ying

**Affiliations:** 1 Department of Obstetrics, Shanghai Key Laboratory of Maternal Fetal Medicine, Shanghai Institute of Maternal-Fetal Medicine and Gynecologic Oncology, Shanghai First Maternity and Infant Hospital, School of Medicine, Tongji University, Shanghai, China; 2 Department of Obstetrics, Shidong Hospital affiliated with the University of Shanghai for Science and Technology, Shanghai, China; 3 Department of Clinical Research Center, Shanghai Key Laboratory of Maternal Fetal Medicine, Shanghai Institute of Maternal-Fetal Medicine and Gynecologic Oncology, Shanghai First Maternity and Infant Hospital, School of Medicine, Tongji University, Shanghai, China

**Keywords:** Generalized estimating equations (GEE), Group-based trajectory model (GBTM), Growth discordance, Preeclampsia, Twins

## Abstract

A retrospective cohort study with 4396 twins who registered before 13 gestational weeks and delivered between January 2013 and December 2020 at Shanghai First Maternity and Infant Hospital, China, was conducted to clarify causal associations between inter-twin estimated fetal weight discordance and preeclampsia. Ultrasound measurements of fetal biometry were collected until the confirmation of preeclampsia diagnosis or the termination of pregnancy (when preeclampsia did not occur). Inter-twin discordance was divided into binary variables using cut-offs of 10%, 15%, and 20%. The associations between inter-twin discordance and preeclampsia were analyzed using generalized estimating equations and group-based trajectory modeling methods. The incidence of preeclampsia was 13.9%, among which 21.8% of cases were diagnosed at early onset and 55.3% at a severe stage. Inter-twin discordance based on estimated fetal weight during pregnancy was positively associated with preeclampsia. The associations were robust and constant by treating the discordance as continuous and binary. Two groups, the stable trajectory group, including 92% of participants, and the changing trajectory group, including 8% of participants, were divided according to the group-based trajectory models. Compared with the stable trajectory group, the risk of developing preeclampsia in the changing trajectory group increased by 50.3% (OR = 1.502, 95%CI: 1.073, 2.105). Subgroup analysis showed positive association primarily in early-onset preeclampsia (OR: 3.859, CI: 2.293, 6.494) and severe preeclampsia (OR: 1.896, CI: 1.264, 2.844) subgroups. These findings can provide a direction to reduce the incidence of preeclampsia in twin pregnancies, considering growth discordance as a high-risk factor in clinical practice.

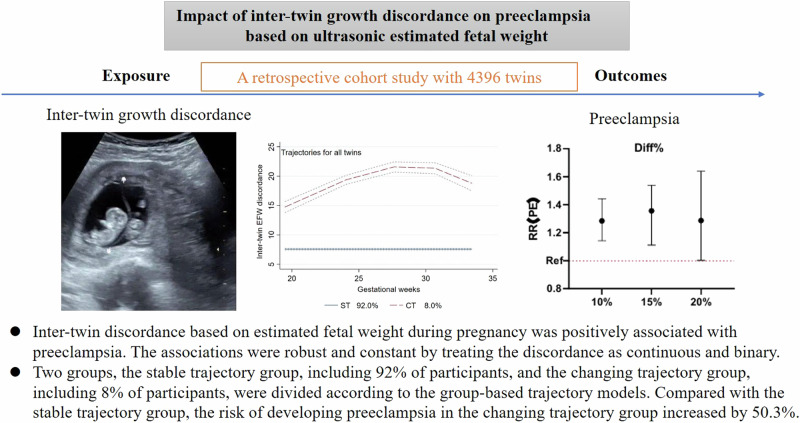

## Introduction

Preeclampsia (PE) affects 2–8% of all pregnant women and is characterized by the presence of hypertension and end-organ dysfunction [1]. According to the American College of Obstetricians and Gynecologists [2, 3], the incidence of PE is three to four-fold higher in twin pregnancies than in singleton pregnancies. PE is among the main reasons for maternal mortality among twin pregnancies and warrants more attention [4].

The pathophysiology of PE is partly caused by abnormal placentation and the woman’s genetic and immunologic predisposition [5]. Defective deep placentation and, subsequently, reduced placental perfusion are the main pathogenesis of PE [6]. Increased PE risk and prevalence in twin pregnancies may be associated with a larger placental mass [1, 7, 8]. Regarding to the recent advances in assisted reproductive techniques (ART), twin and multiple pregnancies have increased during past years (18.9 to 33.3 per 1000 births) [9]. Twin pregnancies increase the complexity of the fetoplacental microenvironment and the risk of adverse factors, especially in inter-twin growth discordance (ITGD), compared with singleton pregnancies [10].

ITGD is the percentage difference in estimated or actual weight between fetuses in a twin pregnancy [10, 11]. Our previous study [12] showed that the prevalence of PE increased significantly in cases of ITGD, defined as the percentage difference in birth weight >20%. However, birth weight inter-twin discordance has limitations for preventing PE [13, 14]. Several studies have reported the association between ITGD and PE [15, 16] using longitudinal estimated fetal weight discordance across gestation, along with an increased risk of PE and adverse pregnancy outcomes among women with ITGD. However, such studies have ignored the chronological sequence of ultrasound measurements and the occurrence of PE. Some measurements are conducted after the diagnosis of PE. The causal relationship between the estimated weight of the fetus after the diagnosis of PE and the association between PE is unclear, so the evidence is controversial.

Therefore, we conducted a study to investigate the association between various degrees of ITGD and PE (including subtypes of PE), utilizing ultrasound assessments of fetal biometry measured before the diagnosis of PE to clarify the causal association between ITGD and PE.

Point of view

- Clinical relevance
  Inter-twin growth discordance, based on estimated fetal weight, may serve as a potential indicator for identifying women at risk of developing preeclampsia, offering valuable clinical predictive information for managing their pregnancies.
- Future direction
  Research into the predictive effectiveness of inter-twin growth discordance for preeclampsia, along with its underlying mechanisms, is necessary.
- Consideration for the Asian population
  Controversies remain regarding the genetic differences in preeclampsia between Europe and Asia. Further investigation is needed to understand how these genetic variations influence the associations between inter-twin growth discordance and preeclampsia.

## Methods

### Study design and population

This was a retrospective cohort study with 204830 pregnant women who delivered infants (5941 twins) between January 2013 and December 2020 at Shanghai First Maternity and Infant Hospital (a tertiary hospital) in Shanghai, China. Pregnant women with the following conditions were excluded: 1) intrauterine fetal death or delivery before 24 gestational weeks; 2) multifetal pregnancy reduction; 3) severe fetal structural or chromosomal abnormalities; 4) complicated twin birth such as twin-to-twin transfusion syndrome, twin-twin anemia-polycythemia sequence, 5) presence of pre-pregnancy severe diseases including hypertension, diabetes or kidney and autoimmune diseases, and 6) those who registered after 13 gestational weeks. Finally, 4396 twin pregnancies were included in the analysis. This study was approved by the hospital’s Human Ethics Committee (registration number: 1KS18118).

### Data collection

All data, including fetal biometric measurements, information on maternal characteristics, medical history, and pregnancy outcomes, were retrieved from the electronic medical records system in our hospital and prospectively entered into a database by research nurses. Data were cross-checked retrospectively by the researchers against the sources (medical records). Repeated ultrasound measurement data, including head circumference (HC), abdominal circumference (AC), femur length (FL), and biparietal diameter (BPD) were measured before the diagnosis of gestational hypertension (GH) or PE for pregnant women who subsequently developed GH or PE. Repeated ultrasound measurement data throughout the pregnancy was selected for those that subsequently remained normotensive.

Gestational age (GA) at the time of ultrasound examinations was determined based on the last menstrual period; the pregnancy was confirmed by ultrasound in the first trimester, and a follow-up was conducted following the relevant guidelines [17, 18].

### Clinical definitions and outcomes

#### Definitions of ITGD

We employed the most accurate EFW, the standard Hadlock III formula, defined as log10 (EFW) = 1.326 − 0.00326 × AC × FL + 0.0107 × HC + 0.0438 × AC + 0.158 × FL, where HC, BPD, AC, and FL are HC, BPD, AC, and FL in centimeters [19]. Currently, no universal cut-offs of ITGD exist; however, researchers commonly use 10%, 15%, and 20% [10]. We classified ITGD as a binary variable.

#### Definitions of PE and subtypes of PE

Preeclampsia was diagnosed based on the American College of Obstetricians and Gynecologists (ACOG) criteria [18]; that is, both blood pressure and proteinuria criteria needed to be fulfilled.

##### Blood pressure criteria

Greater than or equal to 140 mm Hg systolic or greater than or equal to 90 mm Hg diastolic on two occasions at least 4 hours apart after 20 weeks of gestation in a woman with previously normal blood pressure; greater than or equal to 160 mm Hg systolic or greater than or equal to 110 mm Hg diastolic, and hypertension could be confirmed within a short interval (minutes) to facilitate timely antihypertensive therapy

##### Proteinuria criteria

Greater than or equal to 300 mg per 24 h urine collection (or this amount extrapolated from a timed collection) or protein/creatinine ratio greater than or equal to 0.3 mg/ml; Dipstick reading of 1+ (used only if other quantitative methods were not available).

In the absence of proteinuria, preeclampsia was diagnosed as hypertension along with thrombocytopenia (platelet count less than 100,000/microliter), impaired liver function (elevated blood levels of liver transaminases to twice the normal concentration), the new development of renal insufficiency (elevated serum creatinine greater than 1.1 mg/dl or a doubling of serum creatinine in the absence of other renal disease), pulmonary edema, or new-onset cerebral or visual disturbances

Pregnancy with any of the following findings was defined as severe PE (the remaining were cases of mild PE)

Systolic blood pressure of 160 mm Hg or higher, or diastolic blood pressure of 110 mm Hg or higher on two occasions at least 4 h apart while the patient was advised bed rest (unless antihypertensive therapy is initiated before this time)

Thrombocytopenia (platelet count less than 100,000/microliter)

Impaired liver function indicated by abnormally elevated blood concentrations of liver enzymes (to twice normal concentration), severe persistent right upper quadrant or epigastric pain unresponsive to medication and not accounted for by alternative diagnoses or both

Progressive renal insufficiency (serum creatinine concentration greater than 1.1 mg/dl or a doubling of the serum creatinine concentration in the absence of other renal disease)

Pulmonary edema

New-onset cerebral or visual disturbances

Additionally, PE was further divided into early-onset (onset at <34 + 0 weeks of gestation) and late-onset (onset at ≥34 + 0 weeks of gestation) according to the onset of gestational weeks [20].

#### Definitions of GH

GH is blood pressure elevation after 20 weeks of gestation without proteinuria or the aforementioned systemic findings [18].

#### Outcomes

The primary outcome was the incidence of preeclampsia, and the secondary outcome was the incidence of GH and different types of preeclampsia, including severe, mild, early-onset, and late-onset preeclampsia.

### Statistical analysis

Statistical analyses were performed using the Stata software (17.0 MP-Parallel Edition). We described the characteristics of the participants in four groups in terms of frequencies, N (%), for categorical variables and mean ± standard deviation for continuous variables, including the total participants, normotensive pregnancies, GH and PE, EFW for each fetus (continuous) and the degree of ITGD (continuous and categorical) based on ultrasound measurements. The association between different degrees of ITGD (continuous, 10%, 15%, and 20%) and the risk for PE (early or late and mild or severe) were analyzed by generalized estimating equations after adjusting for maternal age, gravidity, parity, pre-pregnancy body mass index (kg/m^2^), ultrasound GA, use of assisted reproductive technology, and presence of gestational diabetes mellitus. Subgroup analysis was conducted using chorionicity in the above models.

Group-based trajectory modeling (GBTM) identifies groups of individuals with similar, inter-related, temporal trajectories of one (single-trajectory model) or many (multi-trajectory model) measurements over time [21–23]. The assumption is that markedly distinct latent trajectory groups exist within the population of interest. Herein, it was used to identify unique trajectory groups of ITGD during the entire pregnancy, based on the degree of ITGD (D%) using ultrasound measurement values. GBTM was performed using the “traj” package in Stata [24]. We fitted between one to four trajectories using the “cnorm” model, yielding a probability for each patient belonging to a trajectory group, referred to as the posterior probability for the corresponding group membership. Twins were assigned to the group, corresponding to the highest probability of group membership. Modeling was based on fitness statistics such as the Bayesian Information Criterion, trajectory group membership (no less than 5%), and the average probability of group membership (no less than 70%) [24]. Sensitive analysis was conducted using the same method after excluding monochorionic twins.

## Results

We analyzed a total of 4396 pregnancies. The incidences of GH and PE were 3.1% and 13.9%, respectively. In total, 17% of participants showed GH/PE. Among the participants, 90.7% (3987) were dichorionic (DC) twins, and the remaining were monochorionic (MC) twins. Most women (83.5%) were nulliparous, and 82.0% were under age 35. More than half (55.4%) underwent assisted reproductive technology with an average preconception body mass index of 22.39. The MC group had a shorter gestational age at delivery compared to the dichorionic DC group (all, normotensive, and GH/PE) and showed a decreasing trend with increasing severity of the disease within each chorionicity group. Throughout the pregnancy, the study collected 15217 ultrasound measurements (1–5 per participant) to identify ITGD. The incidences of ITGD were 31.4%, 13.4%, and 5.0%, with 10%, 15%, and 20% cut-off values, respectively. There were significant differences (*p* < 0.05) between the normotensive, GH, and PE groups in terms of ITGD (mean difference, 10% and 15% cut-offs). The more serious the disease was, the higher the ITGD (mean, >10% and >15%) tended to be (Table 1).

**Table 1:** The characteristics of the participant in each group (*n* = 4396)

Characteristics	Total 4396(100.0)	Normotensive, 3651(83.0)	GH^a^, 134(3.1)	PE^b^,611(13.9)	*p*^c^
Maternal age (years), *n* (%)					
≤30	1558(35.4)	1344(36.8)	40(29.9)	174(28.5)	<0.001
31–35	2050(46.6)	1682(46.1)	65(48.5)	303(49.6)	
>35	788(17.9)	625(17.1)	29(21.6)	134(21.9)	
Gravidity, *n* (%)					
1	2315(52.7)	1909(52.3)	71(53.0)	335(54.8)	0.506
≥2	2081(47.3)	1742(47.7)	63(47.0)	276(45.2)	
Parity, *n* (%)					
0	3672(83.5)	3023(82.8)	120(89.6)	529(86.6)	0.011
≥1	724(16.5)	628(17.2)	14(10.4)	82(13.4)	
ART^d^, *n* (%)	2437(55.4)	1956(53.6)	81(60.5)	400(65.5)	<0.001
GDM^e^, *n* (%)	858(19.5)	686(18.8)	38(28.4)	134(21.9)	0.006
Chronicity, *n* (%)					
DC^f^	3987(90.7)	3288(90.1)	129(96.3)	570(93.3)	0.003
MC^g^	409(9.3)	363(9.9)	5(3.7)	41(6.7)	
GA at delivery^h^, Mean ± SD	36.01 ± 1.80	36.06 ± 1.87	36.33 ± 1.13	35.60 ± 1.35	<0.001
GA at delivery, Mean ± SD (in DC) ^i^	36.25 ± 1.57	36.32 ± 1.63	36.39 ± 1.02	35.7 ± 1.22	<0.001
GA at delivery, Mean ± SD (in MC) ^i^	35.30 ± 1.80	35.36 ± 1.81	34.04 ± 1.20	34.86 ± 1.71	<0.001
Birthweight Large, Mean ± SD	2493.72 ± 442.67	2504.93 ± 449.45	2542.56 ± 335.48	2416.02 ± 413.86	<0.001
Birthweight Small, Mean ± SD	2433.27 ± 454.35	2453.79 ± 446.38	2443.36 ± 409.35	2308.41 ± 431.72	<0.001
Pre-pregnancy BMI (kg/m^2^), Mean ± SD	22.39 ± 3.14	22.23 ± 3.02	23.75 ± 3.21	23.05 ± 3.61	<0.001
Frequency of ultrasonic measurement^j^, (*n* = 15217)					
1	4396(28.9)	3651(28.8)	134(28.1)	611(29.5)	0.993
2	3867(25.4)	3223(25.4)	122(25.5)	522(25.2)	
3	3331(21.9)	2784(22.0)	102(21.3)	445(21.5)	
4	2421(15.9)	2014(15.9)	77(16.1)	330(16.0)	
5	1202(7.9)	998(7.9)	43(9.0)	161(7.8)	
Inter-twin growth discordance, Frequency (%)^k^ (*n* = 15,217)					
10%	4779(31.4)	3862(30.5)	162(33.9)	755(36.5)	<0.001
15%	2038(13.4)	1627(12.8)	69(14.4)	342(16.5)	<0.001
20%	766(5.03)	621(4.9)	21(4.4)	124(6.0)	0.088
Difference%, Mean ± SD^j^ (*n* = 15,217)	8.06 ± 6.38	7.95 ± 6.32	8.21 ± 6.52	8.70 ± 6.71	0.002
EFW_Large^l^, Mean ± SD (*n* = 15,217)	993.75 ± 685.87	995.48 ± 687.29	1003.30 ± 703.25	980.92 ± 673.16	0.774
EFW_Small, Mean ± SD (*n* = 15,217)	925.57 ± 640.56	928.76 ± 643.33	931.38 ± 643.71	904.66 ± 622.50	0.981

^a^GH: Gestational hypertension

^b^PE: Preeclampsia

^c^Obtained through Pearson chi-square test in categorical variables and nonparametric test in continuous variables.

^d^ART: Assisted reproduction technologies

^e^GDM: Gestational diabetes mellitus

^f^DC: Dichorionic

^g^MC: Monochorionic

^h^GA: Gestational age

^i^The p values of nonparametric test between the comparison of GA at delivery in DC and MC groups were all below 0.001

^j^The frequency of positive events (inter-twin growth discordance) measured by ultrasound. Each individual may have multiple positive events during the whole pregnancy

^k^It was calculated by the formula: (the estimated fetal weight of the larger fetus)—(the estimated fetal weight of the smaller fetus)/(estimated fetal weight of the larger fetus) *100%

^l^EFW: Estimated fetal weight

Specifically, 21.8% of cases of early-onset PE were diagnosed, and 55.3% were cases of severe PE. The associations between ITGD and the risk of GH or PE are shown in Table 2. Consistent positive associations between ITGD and GH or PE were found using different cut-offs (10%, 15%, and 20%). The strength of association increased with the degree of ITGD (*p* for trend < 0.001), and this trend was observed in both the PE group and the subgroups of early-onset and severe PE. The risk of developing PE increased by 13.3%, 17.3%, and 19% with ITGD cut-offs of 10%, 15%, and 20%, respectively.

**Table 2:** Associations between inter-twin growth discordance and the risk for GH or PE (*n* = 4396)

GH or PE ^a^	*n* (%)	10%		15%		20%		*P* for trend
		OR^b^	95%CI	OR	95%CI	OR	95%CI	
GH or PE	745(17.0)	1.127	(1.015,1.252)	1.149	(1.037,1.272)	1.167	(1.057,1.287)	<0.001
PE	611(13.9)	1.133	(1.012,1.268)	1.173	(1.050,1.311)	1.190	(1.068,1.327)	<0.001
Early PE	133(21.8)	1.395	(1.082,1.798)	1.453	(1.115,1.893)	1.486	(1.154,1.913)	<0.001
Late PE	478(78.2)	1.083	(0.959,1.223)	1.121	(0.995,1.262)	1.135	(1.010,1.276)	0.053
Mild PE	273(44.7)	1.071	(0.918,1.250)	1.155	(0.990,1.349)	1.172	(1.006,1.366)	0.087
Severe PE	338(55.3)	1.188	(1.021,1.381)	1.186	(1.023,1.375)	1.203	(1.042,1.389)	<0.001

^a^GH: gestational hypertension, PE: preeclampsia

^b^Adjusted for maternal age, chronicity, gravidity, parity, pre-pregnancy BMI (kg/m^2^), gestational weeks at ultrasound measurement, the use of assisted reproduction technologies, and gestational diabetes mellitus

**Fig. 1:**
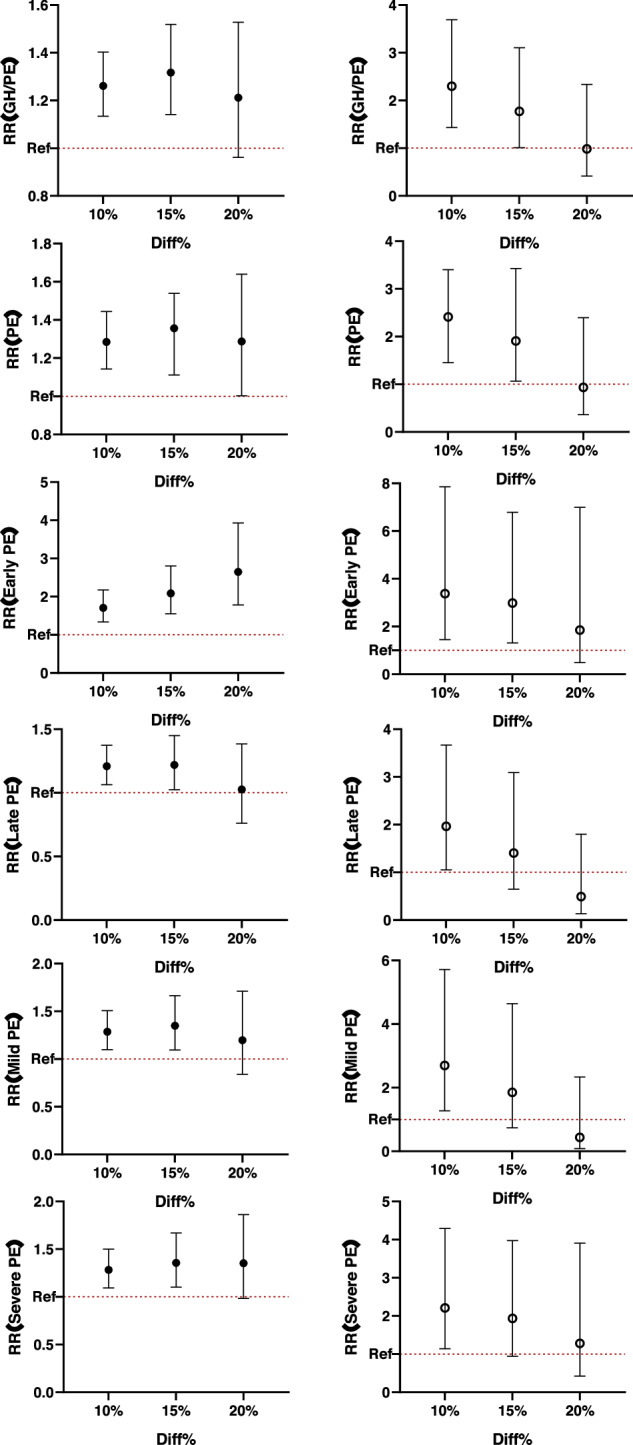
The association between different degrees of inter-twin growth discordance and the risk for GH or PE in DC and MC twins (*n* = 4396). **a** ● for the dichorionic group (DC), ◯ for the monochorionic group (MC). **b** GH: Gestational hypertension; PE: Preeclampsia. **c** Adjusted for maternal age, gravidity, parity, pre-pregnancy BMI (kg/m^2^), Gestational weeks at ultrasound measurement, the usage of assisted reproduction technologies, and Gestational diabetes mellitus. **d** The *p* for trend values were <0.001 (GH/PE), <0.001 (PE), <0.001 (Early PE), 0.044 (Late PE), 0.052 (Mild PE), and <0.001 (Severe PE) in the dichorionic group. **e** The *p* for trend values were 0.012 (GH/PE), 0.012 (PE), 0.004 (Early PE), 0.195 (Late PE), 0.121 (Mild PE), and 0.035 (Severe PE) in the monochorionic group

After stratifying by chorionicity, the associations were retained in both groups. Furthermore, the strength of association (relative risk, RR) between ITGD and PE was stronger in the MC twins than in DC twins (Fig. 1) at ITGD cut-offs of 10% and 15% (Table 3). However, the difference in RR between MC and DC twins was not statistically significant.

**Table 3:** Association between different degrees of inter-twin growth discordance and the risk for GH or PE in DC and MC twins (*n* = 4396)

GH or PE	DC (*n* = 3987)						MC (*n* = 409)	
	10%	15%	20%	*P* for trend	10%	15%	20%	P for trend
	OR^b^ (95%CI)	OR (95%CI)	OR (95%CI)		OR (95%CI)	OR (95%CI)	OR (95%CI)	
GH or PE	1.261(1.134,1.403)	1.317(1.141,1.519)	1.212(0.962,1.528)	<0.001	2.297(1.430,3.690)	1.771(1.011,3.105)	0.985(0.415,2.337)	0.012
PE	1.285 (1.143,1.444)	1.356(1.111,1.540)	1.287(1.002,1.640)	<0.001	2.410(1.452,3.400)	1.909(1.064,3.426)	0.935(0.365,2.395)	0.012
Early PE	1.706(1.337,2.178)	2.085(1.553,2.801)	2.647(1.782,3.931)	<0.001	3.375(1.450,7.855)	2.981(1.309,6.788)	1.850(0.490,6.994)	0.004
Late PE	1.209(1.063,1.374)	1.219(1.025,1.450)	1.027(0.762,1.384)	0.044	1.965(1.053,3.669)	1.404(0.648,3.039)	0.491(0.134,1.797)	0.195
Mild PE	1.286(1.097,1.508)	1.349(1.094,1.664)	1.198(0.839,1.711)	0.052	2.695(1.272,5.711)	1.853(0.739,4.641)	0.435(0.081,2.339)	0.121
Severe PE	1.282(1.095,1.501)	1.356(1.102,1.670)	1.353(0.984,1.860)	<0.001	2.212(1.140,4.292)	1.935(0.943,3.971)	1.283(0.421,3.908)	0.035

^a^GH: gestational hypertension, PE: preeclampsia, DC: Dichorionic group, MC: Monochorionic group

^b^Adjusted for maternal age, gravidity, parity, pre-pregnancy BMI (kg/m^2^), gestational weeks at ultrasound measurement, the use of assisted reproduction technologies, and Gestational diabetes mellitus

**Fig. 2:**
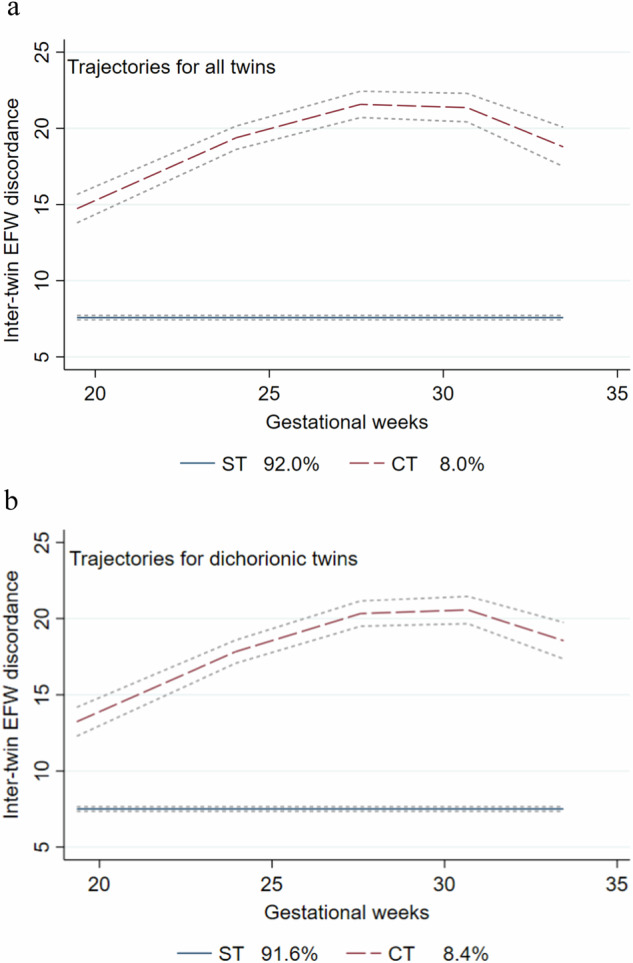
Trajectories for all twins and only dichorionic twins. **a** Blue line: stable trajectory (ST) group including 92% of participants; the red line: changing trajectory (CT) group including 8% participants; the dotted dashed line: 95% confidence interval. **b** EFW: estimated fetal weight. Inter-twin EFW deference was calculated by the following formula: (larger fetal EFW − smaller fetal EFW)/(larger fetal EFW) ×100

For potentially different trajectories of ITGD during pregnancy, GBTM was used to divide the participants into two to four groups based on preset criteria. Two groups of trajectory models were ultimately selected (Fig. S1): the changing trajectory (CT) (ITGD varies throughout pregnancy) with 8% of participants and the stable trajectory (ST) (ITGD remains largely unchanged throughout pregnancy) with 92% of participants (Fig. 2). In CT, the degree of ITGD began (around 20 gestational weeks) at a higher level (nearly 15% or above), increased with the progression of pregnancy, and then decreased after 30 gestational weeks. In contrast, in ST, the degree of ITGD remained stable and at a lower level (below 10%) as pregnancy progressed.

The incidence of PE was 2.8% higher in the CT group (13.7%) than in ST (16.5%), with a greater difference observed in early-onset and severe PE. The risk of developing PE in the CT group increased by 50.3% (OR = 1.502, 95%CI: 1.073, 2.105) compared with the ST group. Subgroup analysis revealed that the positive association was primarily in the subgroups of early-onset PE (OR: 3.859, CI: 2.293, 6.494) and severe PE (OR: 1.896, CI: 1.264, 2.844) (Table 4). Sensitivity analysis showed that after excluding the MC twins, there was little change in ITGD trajectories (Fig. 2). The association between trajectory and PE was unaffected by excluding the MC twins, suggesting a robust association between ITGD and PE.

**Table 4:** Associations between inter-twin growth discordance trajectory and the risk of GH or PE (*N* = 4396)

GH or PE	n (%)		All		DC	
	ST group^a^ 4044(92.0%)	CT group 352(8.0%)	OR^b^	95%CI	OR^c^	95%CI
GH or PE	679(16.8)	66(18.8)	1.387	(1.007,1.910)	1.384	(0.993,1.928)
PE	553(13.7)	58(16.5)	1.5027	(1.073,2.105)	1.496	(1.053,2.124)
Early PE	108(2.7)	25(7.1)	3.859	(2.293,6.494)	3.908	(2.245,6.801)
Late PE	445(11.0)	33(9.4)	1.064	(0.699,1.619)	1.074	(0.698,1.651)
Mild PE	250(6.2)	17(4.8)	1.079	(0.633,1.839)	1.088	(0.630,1.879)
Severe PE	297(7.3)	41(11.6)	1.896	(1.264,2.844)	1.880	(1.229,2.877)

^a^ST group: the stable trajectory group, CT group: the changing trajectory group

^b^Adjusted for maternal age, chorionicity, gravidity, parity, pre-pregnancy BMI (kg/m^2^), gestational weeks at ultrasound measurement, the use of assisted reproduction technologies, and gestational diabetes mellitus

^c^Monochorionic group was excluded

## Discussion

In this large single-center retrospective study on twin pregnancy, consistent positive associations between ITGD and the subsequent GH or PE were found using different cut-offs (10%, 15%, and 20%). The associations were robust and constant by treating the discordance as a binary and continuous variable. Additionally, for pregnant women whose ITGD degree was greater than 15% before 20 weeks of gestation, the risk of developing PE before 24 weeks was 3.9 times of those without ITGD (<10%). The risk of developing severe PE was 1.9 times of those without ITGD (<10%). These findings can provide a direction to reduce the incidence of preeclampsia in twin pregnancies, considering growth discordance as a high-risk factor in clinical practice.

To date, only two relevant studies have been reported [15, 25], and these have identified distinct patterns of ITGD using longitudinal ultrasound measurements throughout pregnancy. One study [15] demonstrated distinct ITGD patterns based on EFW among normotensive, gestational hypertension, and PE, revealing that an ITGD (based on crown-rump length) at 11–14 weeks of gestation was linearly associated with increased risk of PE, especially early-onset PE in DC twins. The other [25] classified pregnancies into four pre-determined patterns based on the timing of onset and the progression of ITGD [25]. However, the aforementioned ITGD patterns are artificially defined, and the patterns in our study are data-driven based on certain parameter selection criteria. Compared with artificially defined patterns, data-driven patterns can better reflect the true trajectory of data. The ITGD trajectories we revealed were divided into only two groups, similar to pattern 1 (ST group) and pattern 2 (CT group) reported by Liran Hiersch et al. [25]. We also observed significant differences in the incidence of PE, especially early-onset PE and severe PE, between the two trajectories.

A Previous study [26] demonstrated that chorionicity is not associated with hypertension disorder of pregnancy, which was consistent with the stratified analysis by chorionicity in our study. Moreover, the group-based trajectory of ITGD in this study remained basically unchanged after excluding MC twins. This suggests that DC twins and MC twins may need to be given equal weight when monitoring the risk of PE due to inconsistent twin growth. However, relevant guidelines recommend that ultrasound examinations should be performed at least once every 2 weeks starting at 16 weeks of gestation, which is twice the recommended frequency of examinations for DC twins.

The pathogenesis of preeclampsia is not fully understood and is complicated by its heterogeneous nature. Preeclampsia comprises various subtypes (e.g., early versus late onset) hypothesized to have different biological pathways leading to the clinical symptoms of preeclampsia [27]. Early onset preeclampsia is associated with an increase in growth-restricted infants and pathological evidence of placental malperfusion [28]. The rate of maternal vascular malperfusion pathology in the placenta of the smaller twin increased with the level of discordance and was the most obvious for discordance ≥25% [29]. The development of growth discordance is a long, slow, and progressive process after the occurrence of placental malperfusion in the smaller fetus, which is similar to SGA and preeclampsia in singleton pregnancy [30]. Twins with growth discordance can align with the pathogenesis, involving both reduced uteroplacental blood supply and increased fetoplacental demands. The biological mechanisms underlying the association between ITGD and PE have not been reported yet. Based on existing research findings and the results of this study, we reasonably speculate that ITGD may be a phenotype underlying the pathological process of PE or that they are all caused by a common cause (e.g., placental malperfusion), and further research is needed to uncover the role of ITGD in the occurrence of PE.

We utilized data from intrauterine EFW of twins during pregnancy to establish an association between ITGD and PE with chronological orders, yielding more clinically relevant results rather than defining ITGD in terms of birth weight. Size discordance at a given point in gestation as a predictor fails to consider important information about the timing and trajectory of ITGD progression [23, 25]. We used longitudinal measurements throughout pregnancy and developed data-driven trajectories for the intrauterine ITGD data, which may have prognostic significance. A series of analyses to elucidate the association between ITGD were conducted. Stratification, trajectory, and sensitivity analyses presented consistent conclusions, implying the robustness of these results. In clinical practice, the focus should be on twin pregnancies with a difference of more than 15% at about 20 weeks of gestation and should be closely followed up ultrasound to evaluate the progress of the difference and choose the appropriate timing of delivery.

However, this single-center study has certain limitations inherent to its retrospective design. Despite rigorous training for sonographers, there may be inconsistencies in the quality control of ultrasound examinations. Although we made efforts to account for possible confounding variables, information on aspirin use was lacking [2], which could result in residual confounding. Furthermore, since this study was exclusively epidemiological, we did not have access to placental pathological findings, emphasizing the need for fundamental and clinical research to gain a more comprehensive understanding of the mechanisms underlying ITGD and PE. Finally, the sample size of MC twins is relatively small (*n* = 409). It may not be sufficient to identify the differences in the association between ITGD and PE among DC twins and MC twins.

ITGD or trajectories can be used as potential proxies for identifying women at risk of developing PE and provide clinical predictive information for the management of their pregnancies. Research on the predictive effectiveness assessment of ITGD for PE and the underlying mechanism is warranted.

### Perspective of Asia

Our study may provide valuable insights for clinical practice aimed at reducing the incidence of preeclampsia in twin pregnancies, particularly by considering growth discordance as a potential risk factor. However, evidence indicates that fetal growth is significantly influenced by ethnicity and nationality. While some previous studies have been multicenter or inclusive of various ethnic groups, leading to a broader range of findings, our study focused solely on Chinese twin pregnancies. This limited sample may be genetically and physically more homogeneous, potentially narrowing observed differences.

Although inter-twin growth discordance is associated with preeclampsia, racial and ethnic disparities are frequently noted in the literature on this condition. Consequently, further research is necessary to investigate the relationship between inter-twin growth discordance and preeclampsia in diverse ethnic groups. Additionally, ongoing debates about the genetic differences in preeclampsia between Europe and Asia highlight the need for more comprehensive studies. Differences in pregnancy metabolic profiles between these populations illustrate how pregnancy characteristics can vary by ethnicity, potentially influencing the incidence of preeclampsia. However, some research suggests that the genetic architecture of preeclampsia may not differ significantly between Central Asian and European populations. Therefore, further exploration is warranted to understand the impact of these genetic differences on preeclampsia trends.

## Supplementary information

Supplementary Figure 1
