# Supplementary material for: Impact of inter-twin growth discordance on preeclampsia: based on ultrasonic estimated fetal weight
Source: Hypertens Res. 2024 Dec 4;48(3):894–903. doi: 10.1038/s41440-024-02027-5 (PMC11879843; doi:10.1038/s41440-024-02027-5)
Supplement: Supplementary file 1 — Supplementary Figure 1 [file 41440_2024_2027_MOESM1_ESM.docx]

**Supplementary Figure 1. The parameters and matrix for the selected trajectory model**
